# Epithelium and stroma from nasal polyp mucosa exhibits inverse expression of TGF-*β*_1_ as compared with healthy nasal mucosa

**DOI:** 10.1186/1916-0216-42-29

**Published:** 2013-04-15

**Authors:** Leonardo Balsalobre, Rogério Pezato, Claudina Perez-Novo, Maria Teresa S Alves, Rodrigo P Santos, Claus Bachert, Luc LM Weckx

**Affiliations:** 1 Department of Otolaryngology, Head and Neck Surgery of Federal University of São Paulo, São Paulo, Brazil; 2 Upper Airway Research Laboratory, Department of Oto-Rhino-Laryngology, Ghent University Hospital, Ghent University, Ghent, Belgium; 3 Department of Pathology, Federal University of São Paulo, São Paulo, Brazil

**Keywords:** TGF-β1, Nasal polyps, Rhinosinusitis, Mucosa

## Abstract

**Objective:**

To evaluate TGF-β_1_ expression in polypoid mucosa (epithelium and stroma) of patients with chronic rhinosinusitis with nasal polyposis (CRSwNP).

**Methods:**

Cross-sectional study with two groups: 17 patients with nasal polyposis and 11 controls. Polyps and normal nasal mucosa were processed by immunohistochemical methods for TGF-β1 visualization. Then, the percentage of TGF-β1 expression in stroma and epithelium was objectively quantified using UT Morph software.

**Results:**

A lower percentage of positive expression was found in the epithelium of CRSwNP patients (32.44%) versus normal controls (55.91%) (p < 0.05), and a higher percentage of positive expression in the stroma of CRSwNP patients (23.24%) versus controls (5.88%) (p < 0.05).

**Conclusion:**

The lower percentage of TGF-β_1_ expression in the nasal epithelium of CRSwNP patients may have an impact on epithelium-directed topical treatments employed in this patient population.

## Introduction

Transforming growth factor beta-1 (TGF-β_1_) is implicated as a key protein in the tissue remodeling process. It stimulates fibrosis (by attracting stromal cells), angiogenesis, and accumulation of extracellular matrix [1,2].

In chronic rhinosinusitis with nasal polyposis (CRSwNP), the remodeling process warrants a closer look, as nasal polyp histology is characterized by diffuse mucosal edema with a lack of extracellular matrix, unlike chronic rhinosinusitis without nasal polyposis (CRS) [3,4].

There is still controversy in the literature as to TGF-β_1_ expression in nasal polyposis. Some authors have demonstrated higher TGF-β_1_ expression in patients CRSwNP than in patients with CRS and controls [5-8], whereas other authors have reported opposite findings, with lower TGF-β_1_ expression in CRSwNP than in CRS and healthy controls [9,10].

There are some reasonable explanations for the discrepancy found in the literature. Firstly, studies have not discriminated between cell types for measurement, nor have they distinguished epithelium from stroma, and, interestingly, experiments normally use inferior turbinate mucosa from healthy subjects as controls for comparison with sinus mucosa from CRS patients.

Another relevant potential confounding factor is the lack of criteria for patient selection, such as no inclusion of washout periods for corticosteroids and antibiotics, or no assessment for atopic or asthmatic status [11,12]. The technique used to evaluate TGF-β_1_ expression can also interfere with study results. We noted a trend toward increased TGF-β_1_ expression in CRSwNP patients when immunohistochemistry (IHC) is employed [13-16].

One factor that limits the reliability of IHC is the use of a semi-quantitative method for evaluation, which is thus subject to investigator bias.

The present study is novel in that we evaluated TGF-β_1_ expression in polyp mucosa of CRSwNP patients by stratifying the mucosa into epithelium and stroma groups and using a purely quantitative method for assessment of IHC slides. Furthermore, mucosa from the ethmoidal bulla was used as a control.

## Material and methods

Patients were recruited at the Department of Otorhinolaryngology, Federal University of São Paulo, Brazil. Samples of ethmoid bulla mucosa from patients without sinus disease who underwent endoscopic hypophysectomy were used as controls (n = 11). Samples from patients with adult nasal polyposis (CRSwNP, n = 17) were obtained during functional endoscopic sinus surgery (FESS) procedures. The diagnosis of sinus disease was based on history, clinical examination, nasal endoscopy, and computed tomography (CT scan) of the paranasal cavities, according to EPOS guidelines [17].

All subjects underwent a skin prick test for common inhalant allergens (*Aspergillus fumigatus*, *Penicillium notatum*, *D. pteronyssinus*, *D. farina*, *Lolium perenne*, *C. herbarum*, *E. mainei*, *T. putrescentiae*, *B. tropicalis*, *B. kulagini*, cat and dog fur) and all tests had to be negative for the subject to be included in the experiment. Other criteria of exclusion were a diagnosis of asthma (based on ventilatory test), aspirin intolerance (based on history), or cystic fibrosis.

All subjects included in the study underwent a washout period of 30 days for corticosteroids and antibiotics.

The study was approved by the Research Ethics Committee of the Federal University of São Paulo, Brazil (registry number 0544/09) and all participants provided written informed consent prior to sample collection.

### Tissue preservation and preparation for staining

Ethmoid bulla mucosa from healthy subjects and nasal polyp tissue from CRSwNP patients were fixated in 10% acetaldehyde for 24 hours at room temperature immediately after surgical removal.

The specimens were preserved in 70% ethanol at 4°C, embedded in paraffin, and cut in a microtome into 4-micron sections, which were then affixed onto Superfrost Plus glass slides (Menzel Glaser, Braunschweig, Germany). The slides were then dried at 60°C for a few hours.

For deparaffinization, the slides were washed successively in xylene (3 times), 100% ethanol (3 times for 5 minutes), and distilled water (for 5 minutes). Antigen retrieval was done as follows: briefly, a staining dish containing citrate buffer (pH 6.0) was pre-heated in a steamer until the temperature reached 95–100°C. The slides were then immersed in the staining dish. The staining dish was cooled at room temperature. Endogenous peroxidase was blocked with hydrogen peroxide prior to hybridization.

Sections were rinsed in distilled water and subsequently in PBS (pH 7.4–7.8), 2 changes, for 2 minutes.

After air-drying, the sections were incubated with mouse anti-human TGF-β_1_ monoclonal primary antibody (Biosource International Inc., Camarillo, USA) and diluted in bovine serum albumin (BSA) 1% buffer overnight.

The standard immunohistochemistry protocol was then initiated: slides were washed in PBS and incubated with the first reagent (KIT LSAB/DAKO, Glostrup Denmark) for 30 minutes. The procedure was repeated once more, with the second reagent (KIT LSAB/DAKO, Glostrup Denmark) replacing the first one. After PBS rinsing, a DAB reaction was carried out (0.06 g DAB, 100 mL PBS, 1 mL H_2_O_2_) for 5 minutes and slides rinsed in running tap water.

The slides were counterstained using Harris’ hematoxylin solution for 3 minutes.

Finally, the slides were rinsed in running tap water, dehydrated, cleared, and set in coverslips with resin.

### Morphometric method

Microscopic examination was conducted by an investigator blinded to diagnosis. An Olympus BX51 microscope (Shinjuku, Japan) equipped with an Oly 200 camera (Olympus America Inc., Center Valley, USA) and a processor (Intel Core 2 DUO, Santa Clara, USA) was used. Images were captured using an Intel Core 2 Duo workstation, a plate reader, and UT Morph version 2.0 software, developed by University of Texas.

Five fields per slide were examined under 400× magnification, starting at the most intensely stained area and moving in sequence to randomly selected adjacent fields.

**Figure 1:**
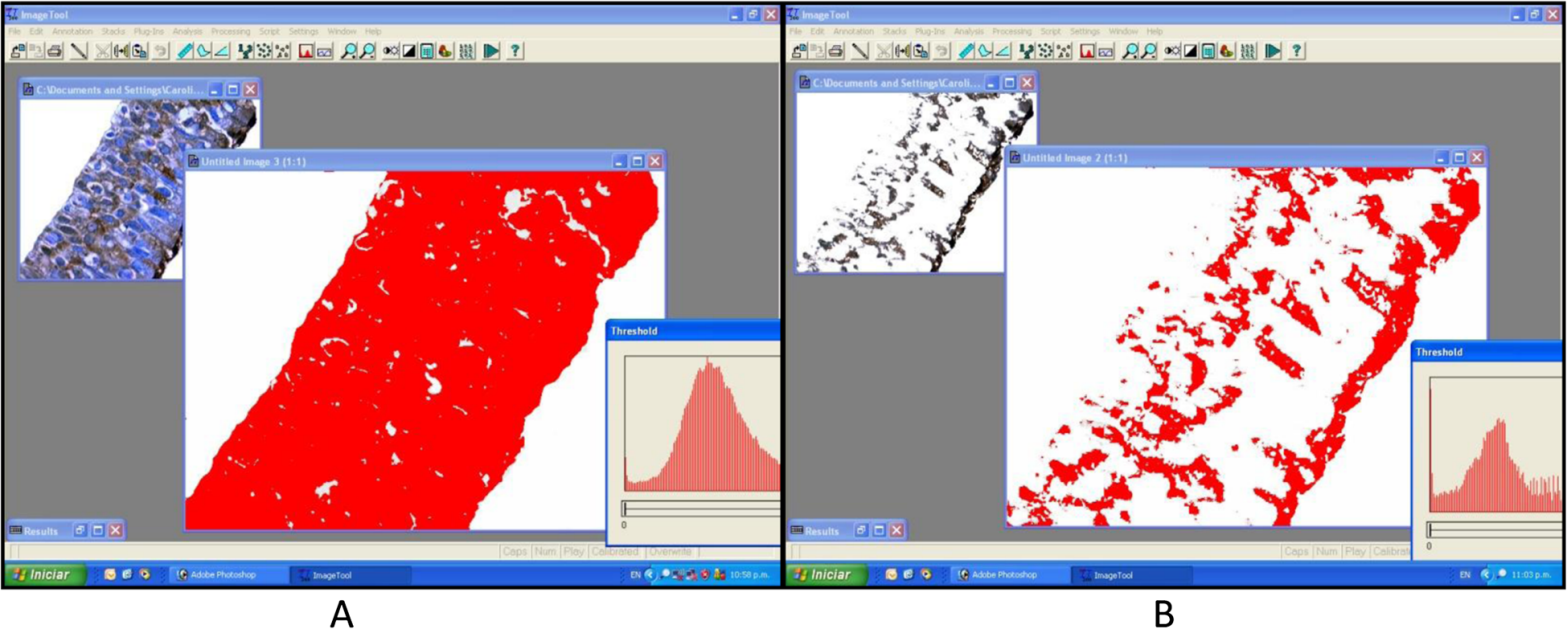
**Digital separation between epithelium and stroma. A**) Total epithelium area (red) measured in UT Morph software. **B**) Area positive for expression of TGF-β_1_ (red).

**Figure 2:**
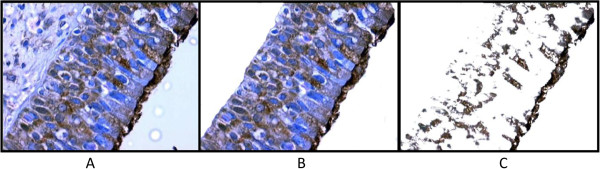
**Assessment of TGF-b1 percentage in the epithelium. A**) Histological section (400× magnification) showing stroma and epithelium (TGF-β_1_ expression shown in brown). **B**) Stroma excluded and epithelium preserved. **C**) Positive TGF-β_1_ expression in epithelium after exclusion of the non-positive area.

UT Morph 2.0 software was used to quantify the percentage of immunostained area (Figure 1). Mucosal images were split into epithelium and stroma using Corel Photo-Paint 9 software (Ottawa, Canada) (Figure 2).

### Statistics

The data obtained were analyzed using SPSS 18 software (IBM Corporation, NY, USA). The Kolmogorov-Smirnov (KS) test was used to assess the normality of distribution. Student’s *t*-test and the Mann-Whitney *U* test (for normally and non-normally distributed data respectively) were used to test for statistically significant between-group differences. *P* values of <0.05 were considered significant.

## Results

### Epithelium

Percent area with positive expression of TGF-β_1_ in the epithelium is demonstrated in Table 1 (16 CRSwNP/11 controls). One CRSwNP slide was excluded due to partial damage of the epithelium; however, the stroma portion was preserved. We found a smaller percent area of TGF-β_1_ expression in the epithelium of CRSwNP patients (32.44%) as compared with controls (55.91%), p < 0.05 (Table 2).

**Table 1:** **Comparison between percent area of TGF-β**_**1 **_**expression in the epithelium, controls versus CRSwNP patients**

**Epithelium**			
**CRSwNP**		**Control**	
1	39,21%	1	41,62%
2	31,50%	2	77,41%
3	13,55%	3	46,93%
4	44,55%	4	24,38%
5	42,13%	5	62,07%
6	46,45%	6	51,53%
7	46,21%	7	47,75%
8	29,14%	8	59,70%
9	47,49%	9	82,24%
10	32,75%	10	86,01%
11	21,10%	11	35,28%
12	21,56%		
13	17,14%		
14	41,68%		
15	15,41%		
16	29.16%		

**Table 2:** **Statistically significant differences in percent area of TGF-β**_**1 **_**expression in the epithelium, controls versus CRSwNP patients**

**Epithelium**			
		**Positive area (%)**	
	**CRSwNP**		**Control**
Mean	32.44		55.91
95% CI	32.06–38.80		42.63–69.18
Standard deviation	11.93		19.76
Minimum	13.56		24.39
Maximun	47.50		86.02

95%: CI, 95% confidence interval.

Student’s *t* test, p= 0.007.

Kolmogrow-Sminov test, polyposis group, p = 0.85; control group, p = 0.99.

### Stroma (submucosa)

Percent area with positive expression of TGF-β_1_ in the stroma is demonstrated in Table 3 (17 CRSwNP/11 controls). We found a greater percent area of TGF-β_1_ expression in the stroma of CRSwNP patients (23.24%) as compared with controls (5.88%), p < 0.05 (Table 4).

**Table 3:** **Comparison between percent area of TGF-β**_**1 **_**expression in the submucosa, controls versus CRSwNP patients**

**Submucosa**			
**CRSwNP**		**Control**	
1	35.07%	1	20.56%
2	12.23%	2	0.00%
3	24.66%	3	0.00%
4	18.53%	4	0.00%
5	19.75%	5	10.80%
6	17.15%	6	0.00%
7	22.16%	7	0.00%
8	25.64%	8	0.00%
9	20.26%	9	15.54%
10	18.00%	10	15.77%
11	44.06%	11	0.00%
12	25.82%		
13	31,50%		
14	34.60%		
15	13.17%		
16	20.64%		
17	11.87%		

**Table 4:** **Statistically significant differences in percent area of TGF-β**_**1 **_**expression in the submucosa, controls versus CRSwNP patients**

**Submucosa**			
		**Positive area (%)**	
	**CRSwNP**		**Control**
Mean	23.24		5.88
95% CI	18.69–27.79		0.1173–11.64
Standard deviation	8.85		8.58
Minimum	11.87		0
Median	20.64		0
Maximun	44.06		22.56
Kolmogorov-Sminov test:	p = 0.41		p = 0.0003*
Mann-Whitney.			
		p = 0.03*	

## Discussion

TGF-β_1_ plays a key role in the remodeling process, contributing to the interstitial matrix formation. There is discrepant information on TGF-β_1_ expression in CRSwNP. Some authors have reported greater expression of TGF-β_1_ in patients with CRSwNP as compared with healthy subjects, [5-8] while other authors found the opposite [9,10].

In the present study, we demonstrated the importance of splitting the nasal mucosa into epithelium and stroma, because in CRSwNP, these cell subsets express TGF-β_1_ differently as compared with healthy nasal mucosa.

Because of the similarities between upper and lower airway mucosa, some authors have compared the remodeling process between these tissues. Bosquet et al. found a thinner basement membrane with fewer elastase-positive cells in nasal mucosa when compared to bronchial mucosa [18]. The authors also demonstrated that nasal epithelium disruption is less extensive than that observed in the lungs.

The importance of epithelial integrity and the role of TGF-ß on the remodeling process were demonstrated by Holgate et al., who found that epithelial injury results in increased production and release of TGF-ß [19].

The fact that the percentage of epithelium expressing TGF-β_1_ was lower in patients with CRSwNP than in healthy subjects in our study supports the hypothesis that the nasal mucosa may offer less resistance to edema during the inflammatory process of nasal polyposis [4]. Accordingly, Li X et al. demonstrated decreased expression of collagen in CRSwNP versus CRS, and suggested TGF-β as a main switch for different remodeling patterns in sinus disease [3]. Van Bruaene et al. also corroborated these findings, showing low concentrations of TGF-β_1_ and collagen and low expression of TGF-β receptor II and receptor III mRNA in patients with CRSwNP as compared with controls [20].

Another factor that might contribute to different TGF-β_1_ expression in nasal polyps is the technique used for evaluation. Methods such as ELISA or even PCR applied to nasal tissue homogenates provide a better picture of total load and transcription of TGF-β_1_ respectively. However, they fail to localize the distribution of TGF-β_1_; consequently, IHC is used.

IHC has tended to show increased TGF-β in patients with CRSwNP versus controls [3,6,16,18], but this technique is usually employed with semi-quantitative methods for image assessment [7,13]. In our study, we used a quantitative method to evaluate the percentage of area stained by TGF-β_1_. The weakness of this method is that it does not allow assessment of the intensity of staining.

Finally, we must stress the importance of other variables that could lead to erroneous interpretations, such as use of antibiotics, anti-inflammatory or immunosuppressant agents, as some of these drugs have shown anti-inflammatory effects in the nasal mucosa [11,12]. Furthermore, failure to distinguish atopic or aspirin tolerance status during patient selection can produce misleading results. In this study, the minimum washout period for antibiotics and corticosteroids was 30 days, and only patients who had negative skin prick tests and no diagnosis of asthma were included.

The most novel contribution of this experiment was perhaps the use of ethmoidal mucosa from patients undergoing hypophysectomy as controls to be compared with nasal polyp tissue. In contrast, most studies use inferior turbinate tissue from patients undergoing rhinoplasty or septoplasty as controls for comparison with nasal polyps [13,15,16].

In conclusion, we found a relative decrease in percent area of TGF-β_1_ expression in CRSwNP epithelium and a relative increase in percent area of TGF-β_1_ expression in the CRSwNP stroma when compared to healthy nasal mucosa. These findings could have an impact on topical treatments for patients with CRSwNP.

## Competing interests

The authors declare that they have no competing interests.

## Authors’ contributions

RP, MTSA, LB have made substantial contributions to conception and design. LB, LLMW were involved in data collection. LB, RP, MTSA were involved in statistical analysis. RP, CB, CPN, MTSA, LLMW, LB were involved in drafting of the manuscript. CB, CPN, LLMW were involved in revising the manuscript critically for important intellectual content. All authors have given final approval of the version to be published. All authors read and approved the final manuscript.
